# Establishing regions of interest of the lower leg and ankle for perioperative volumetric assessment with a portable 3D scanner in orthopedic and trauma surgery – a pilot study

**DOI:** 10.1186/s13047-023-00684-2

**Published:** 2023-12-05

**Authors:** Roman Taday, Erik Schiffner, Sebastian Viktor Gehrmann, Lena Marie Wilms, Robert Alexander Kaufmann, Joachim Windolf, David Latz

**Affiliations:** 1 Department of Orthopedic and Trauma Surgery, University Hospital Düsseldorf, Moorenstraße 5, 40255 Düsseldorf, Germany; 2 Department of Orthopedic and Trauma Surgery, Katholisches Karl- Leisner Klinikum, Albersallee 5-7, 47533 Kleve, Germany; 3 Department of Radiology, University Hospital Düsseldorf, Moorenstraße 5, 40255 Düsseldorf, Germany; 4 Department of Orthopedic Surgery, University of Pittsburgh Medical Center, 3471 Fifth Avenue, Pittsburgh, PA 15213 USA

**Keywords:** 3 D volumetric analysis, Three-dimensional imaging, Artec EVA, Ankle fracture, Soft tissue management

## Abstract

**Background:**

Soft tissue swelling assessment benefits from a reproducible and easy to use measurement method. Monitoring of the injured lower extremity is of clinical import during staged soft tissue management. Portable 3D scanners offer a novel and precise option to quantify and contrast the shapes and volumes of the injured and contralateral uninjured limbs. This study determined three regions of interest (ROI) within the lower extremity (lower leg, ankle and foot), that can be used to evaluate 3D volumetric assessment for staged soft tissue management in orthopedic and trauma surgery.

**Methods:**

Twelve healthy volunteers (24 legs) were included in this cohort study. Scans of all three ROI were recorded with a portable 3D scanner (Artec, 3D scanner EVA) and compared between the right and left leg using the software Artec Studio (Arctec Group, Luxemburg).

**Results:**

Mean volume of the right leg was 1926.64 ± 308.84 ml (mean ± SD). ROI: lower leg: 931.86 ± 236.15 ml; ankle: 201.56 ± 27.88 ml; foot: 793.21 ± 112.28 ml. Mean volume of the left leg was 1937.73 ± 329.51 ml. ROI: lower leg: 933.59 ± 251.12 ml; ankle: 201.53 ± 25.54 ml; foot: 802.62 ± 124.83 ml. There was no significant difference of the overall volume between right and left leg (*p* > 0.05; overall volume: △ difference: 29.5 ± 7.29 ml, *p* = 0.8; lower leg: △ difference: 21.5 ± 6.39 ml, *p* = 0.8; ankle: △ difference: 5.3 ± 2.11 ml, *p* = 0.4; △ difference: 16.33 ± 4.45 ml, *p* = 0.8.

**Conclusion:**

This pilot study defines three regions of interest of the lower leg and demonstrates no difference between the right and left side. Based on these ROI, further studies are needed to evaluate the clinical applicability of the scanner.

## Introduction

Management of lower extremity orthopedic trauma is substantially influenced by the soft tissue swelling [1]. Optimizing the time interval between trauma and open reduction and internal fracture fixation particularly when treating unstable ankle fractures is imperative as excessive swelling is highly correlated with soft tissue complications, such as infection, osteomyelitis, skin necrosis and wound dehiscence [2]. Mitigation strategies delay definitive surgery by employing a two- stage algorithm that begins with closed reduction and external fixation until the soft tissue envelope is deemed acceptable [3]. The perioperative assessment of when to perform definitive treatment is still highly subjective and relies on the surgeon’s experience. There exists a need to define an objective baseline of bilateral soft tissue envelopes so as to assist with the timing of definitive surgical treatment [2]. Valid tools in the assessment of soft tissue volumes are tape measurements (e.g. figure of eight technique) and water displacement methods. These techniques either do not give an accurate representation of swelling throughout the entire lower leg, ankle, and foot or are too time consuming and expensive for use in the clinical practice [4, 5]. There is currently no objective and reliable method to assess swelling of soft tissue around the ankle after trauma. There are many strategies for decongestive treatment of critical soft tissue mantle after ankle injuries for which there is no clear evidence [6, 7]. To make decongestive treatment strategies comparable, a reliable and valid method to measure soft tissue swelling of the injured ankle is needed. A portable three- dimensional (3D) scanner can achieve efficient, objective and reproducible volume measurements and demonstrates a high correlation with tape measurement & water displacement methods [8, 9]. In order to make volume changes of the ankle’s soft tissue mantle comparable throughout a test population a side-by-side comparison is needed. Therefore, it is important to find regions of interest of the ankle which are valid and reliable for a side-by-side comparison. The aim of this pilot study was to characterize three regions of interest that are suitable for side-to-side comparison of both legs with a portable 3D scanner (Artec 3D scanner EVA) in healthy probands.

## Materials and methods

### Population

Twelve healthy volunteers (24 legs, 7 women, 5 men) were included in this study. Participants who documented injuries or any other functional, musculoskeletal disorders regarding knee, lower leg, ankle or foot were excluded from the study. Each subject completed a standardized questionnaire (age, height, weight, gender, leg dominance). Informed written consent was obtained from all participants prior to the procedure. The study was performed according to the guidelines provided by the Declaration of Helsinki and was approved by the ethical committee of the university of Düsseldorf (Ethikkommission an der Medizinischen Fakultät der Heinrich-Heine-Universität Düsseldorf; APPROVAL NUMBER 2019 − 475).

### Image processing and 3D analysis

**Fig. 1:**
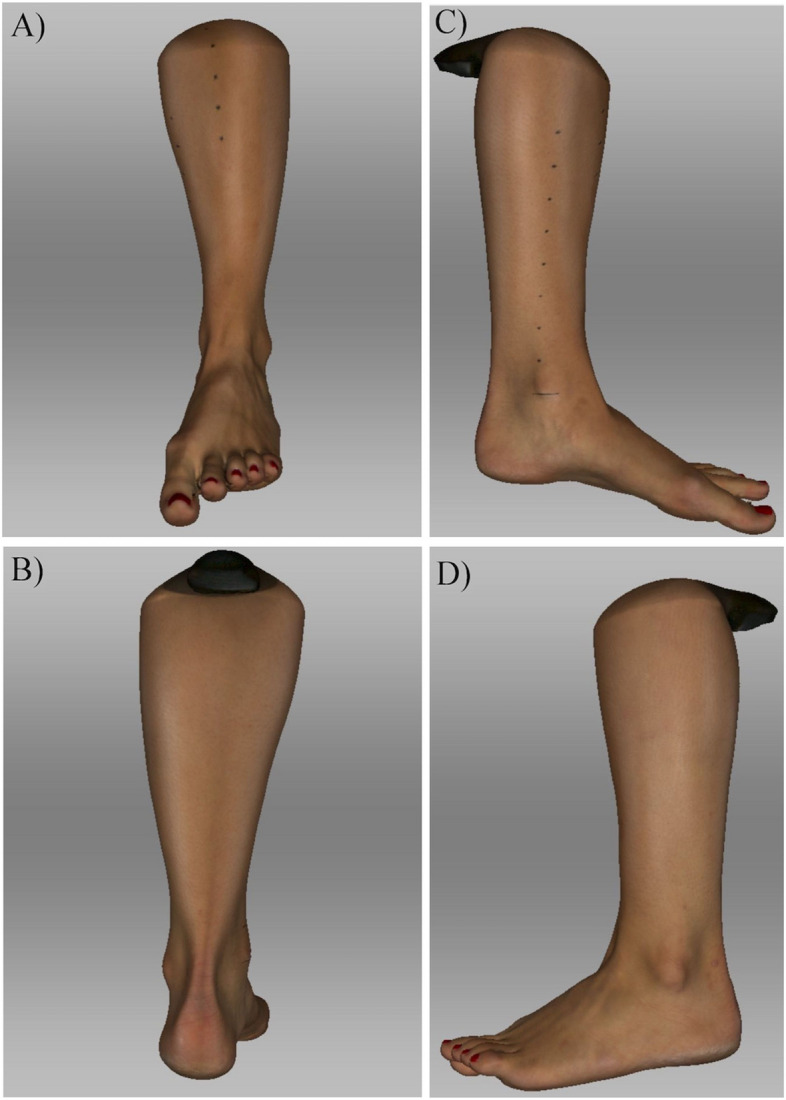
Color texturized 3D pictures of a left leg. (**A**) a.p.; (**B**) p.a.; (**C**) medial (with marked anatomical landmarks); (**D**) lateral after the scanning process

The portable handheld scanner Artec EVA (Artec Group, Luxemburg) uses a structured light triangulation technique to create a 3D mid-size model. The scanner recognizes and records the topography within the region of interest (ROI) using normal visible light, without any harmful radiation by two cameras. A third camera, which is in the middle of the scanner, receives texture information using hybrid geometry and color tracking methods. Artec EVA can take up to sixteen 3D pictures per second without prior calibration. The pictures are automatically processed by Artec Studio 13 software (Arctec Group, Luxemburg). After the scanning process, all pictures and texture information are fused and merged by the software to create a color texturized 3D scan with a resolution of 0,2 mm (Fig. 1). The scan is received in a STL file and is exported to a computer as a Joint Photographic Experts Group File Interchange Format (.jpg) together with texture mapping information inside a Material Template Library file (.mtl). The scanner is an approved and validated instrument in various technical fields, medical engineering and science [8, 10–13].

### Study protocol and scanning procedure

**Fig. 2:**
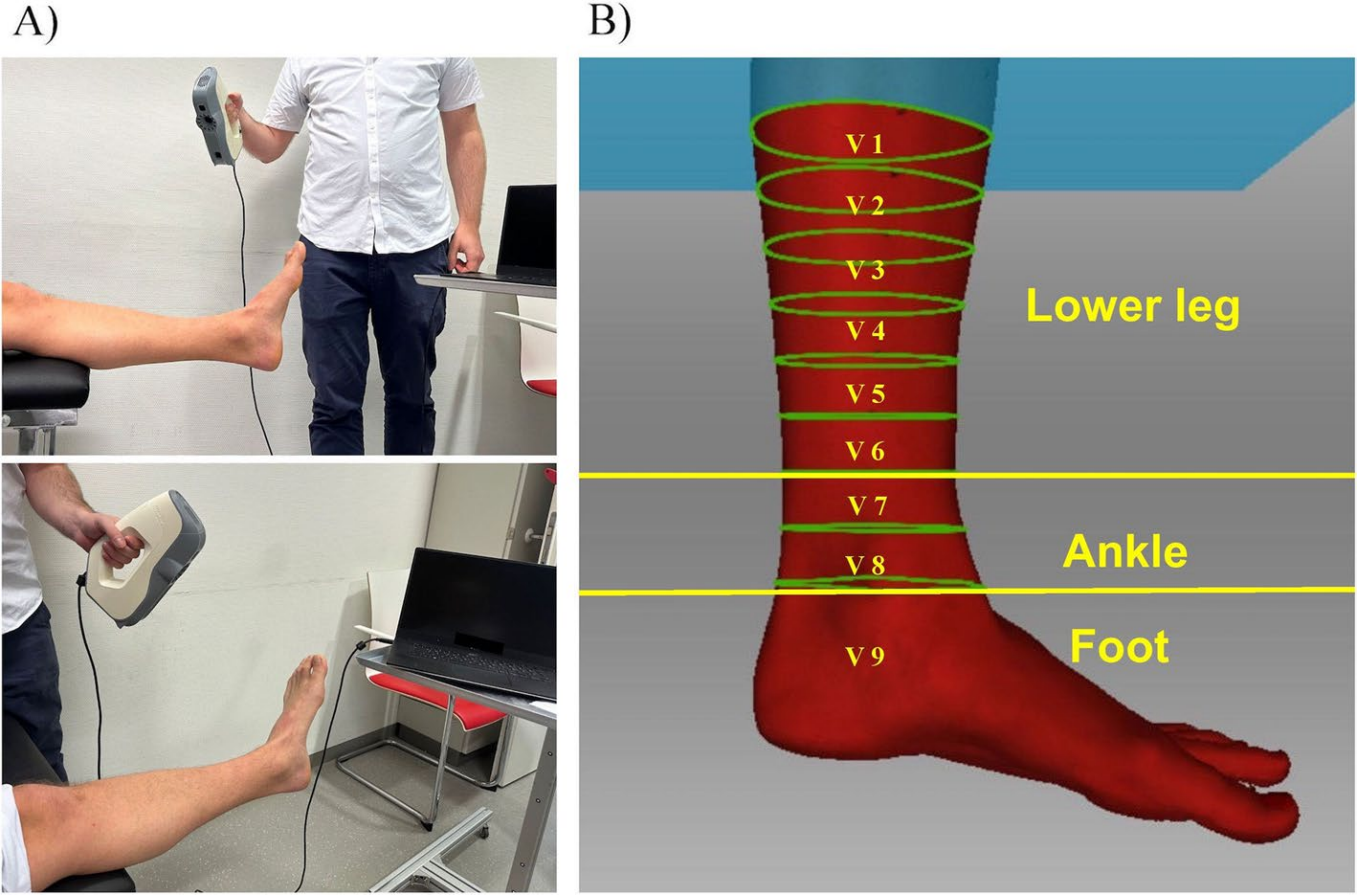
Outline of scanning process and determination of the ROI.  **A** Image of the 3D scanning process. The scanner is hand held and connected to a notebook, which is resting on a mobile cart. The scanner is equipped with a rechargeable battery, so a power outlet is not necessary as a power source. Thus, the scanner can be guided 360° around the lower extremity by the examiner. **B** Determination of three regions of interest (ROI) subdivided in segments of 2.5 cm: Lower leg : 15 cm proximal from ROI ankle (V1 to 6). Ankle : 5 cm proximal from the tip of the medial malleolus (V7 to 8; framed by yellow lines). Foot : whole foot distal to the malleolus fork (Volume 9). Illustration by the authors with Artec Studio 13 software program Version 13, Artec Group, Luxembourg

All scans were recorded by one examiner with a hand held 3D scanner (Artec, Modell EVA). The scans took place in the same room, with an ensured constant ambient temperature trough air conditioning. For the measurement, the light in the room is dimmed to avoid disturbing light rays. The scanner does not need to be calibrated prior scanning. Anatomical landmarks were indicated with a marker. For an objective, reliable and reproduceable assessment of volume, the medial and lateral malleolus were established as anatomical landmarks with a previously described method that enjoys high intra- and interobserver reliability (Fig. 1) [4, 14–17]. The circumferences were marked and subdivided in segments of 2.5 cm (Nine Volumes V1 to 9; Fig. 2). The distance of the measurement area extended 20 cm proximal to the malleolus fork and distally included the entire foot. The dimensions of the ROI were chosen to cover typical surgical approaches. The volunteers were seated and their full extended legs were placed on a rest table with an exposed ankle at the level of the heart. During the measurement, the ankle was maintained in a neutral position regarding eversion and inversion with the distal 30 cm of the leg beyond the support and the ankle in a 90° angle to the leg. For the scanning process, a visible light network is generated by the scanner, which scans the topography of the ankle, while the examiner moved the scanner around the exposed ankle until the entire ROI were completely recorded by 360 degrees (Fig. 2). The procedure was repeated for the other leg so that both legs were measured. All volunteers were instructed not to move during the scans. The ideal distance (approx. 1 m) to scan was determined by the distance adjustment indicator within the Artec Studio 13 software program (Version 13, Artec Group, Luxembourg).

### Statistical analysis

Statistical analysis was performed using Statistical Package for Social Sciences (SPSS). For normality assessment, the D’Agostino-Pearson normality test was used. To compare the volume differences between the left and right leg we performed Wilcoxon-Mann-Whitney signed-rank test. *P*- values ≤ 0.05 were considered a significant difference. An a priori power analysis (G*Power Version 3.0.10, Franz Faul, University of Kiel, Germany) resulted in a sample size of 10 for a power of 80% with a *p* value of 0.05 determining significance.

## Results

Seven women and five men formed the collective of this study (mean age 27.1 ± 3 years). The participants cohort showed a mean weight of 70 ± 13 kg and a mean height of 171 ± 8.8 cm. Overall volumes ranged from 1210 ml to 2645 ml. The duration of each 3D- scan of both legs was 5.1 ± 2 min.

### Comparison of left and right leg

**Fig. 3:**
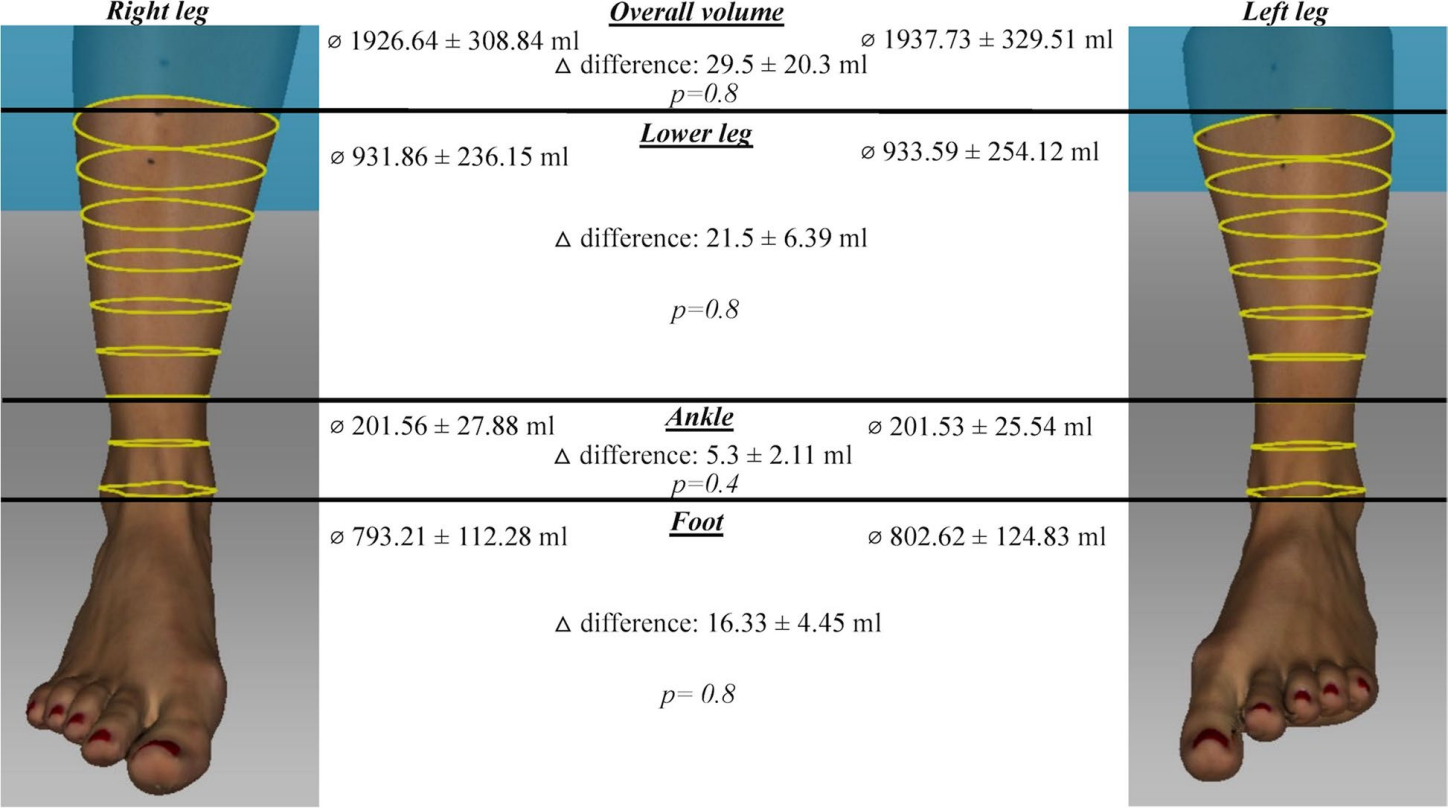
Side- by- side comparison of both legs.  3D a.p. view of an example proband’s right and left leg (by Artec Studio 13 software program Version 13). Yellow rings marking the volume segments of 2.5 cm. The black lines highlight the boundaries between the respective regions of interest. Comparison of both legs indicating no significant volume difference (*p* > 0.05) between the overall volume and respective ROI of all probands

 Overall ankle volume and volume of the ROI were compared between left and right leg (Fig. 3). Mean volume of proband’s right leg was 1926.64 ± 308.84 ml (mean ± SD). ROI right: lower leg: 931.86 ± 236.15 ml; ankle: 201.56 ± 27.88 ml; foot: 793.21 ± 112.28 ml. Mean volume of proband’s left leg was 1937.73 ± 329.51 ml. ROI left: lower leg: 933.59 ± 251.12 ml; ankle: 201.53 ± 25.54 ml; foot: 802.62 ± 124.83 ml. D’Agostino-Pearson normality test showed that the data was not normally distributed. Wilcoxon- Mann- Whitney test was performed to examine a difference between the left and the right ankle. Test results: overall volume: △ difference: 29.5 ± 7.29 ml, *p* = 0.8; lower leg: △ difference: 21.5 ± 6.39 ml, *p* = 0.8; ankle: △ difference: 5.3 ± 2.11 ml, *p* = 0.4; △ difference: 16.33 ± 4.45 ml, *p* = 0.8. No significant volume differences between supporting and free leg were found (*p* > 0.05).

## Discussion

Clinical soft tissue characterization when managing severely injured limbs remains challenging and has a large subjective component based on anecdotal experience of the treating surgeon, which may lead to variable treatment algorithms that lack consensus opinion [18, 19]. Inaccurate assessment may affect the timing of surgery and is highly correlated with soft tissue complications and longer hospitalization [2]. Decreasing the subjective nature of evaluation has led to different measurements of circumference and volume such as bioelectrical impedance, computer-aided systems (Vectra 3D Imaging), disc method, tape measurement as well as water displacement methods [12, 13, 20, 21]. Water displacement and tape measurement represent reliable tools to measure limb swelling [8, 22, 23] and yet water displacement cannot be used with open wounds or with external fixation [4]. Moreover, the water displacement method provides no information about the shape of the injured extremity [8, 21, 22]. Figure of eight measurement is a reliable, time and cost efficient method for measuring swelling around the ankle [4] but is not employed above the ankle and just like circumferential band measurements is poorly suited when managing open wounds in severely injured extremities [5]. An ideal method for volume assessment in injured limbs should be valid, reliable, non- invasive, expedient and without radiation exposure. With a resolution of 0,1 mm the portable Artec Eva 3D- Scanner offers these advantages, and has shown significant correlation to the water displacement method with an identified mean error of only 1.4% in previous studies [9, 10, 24–27]. Due to high resolution and three- dimensional representation, swelling conditions of the lower extremities can be assessed even in the case of concomitant open wounds. An additional advantage over the water displacement method, is the ability of the Artec Studio 13 Software (Version 13, Artec Group, Luxembourg) to edit out external fixation. A splint, however, must be removed before scanning.

An important component in the management of soft tissue pathology is the ability to compare to the contralateral healthy limb and detect volume differences in defined regions of interest between the injured and uninjured lower extremity. Portable 3D scanners are capable of quickly examining specific ROI, and quantifying the change in shape and volume [24]. We chose the anatomical landmarks for the ROI with the highest interobserver and intraobserver reliability according to previous studies [4, 14–17]. The average length of the human soleus muscle as 290–380 mm at ankle joint angles of 0 to 35 degrees (i.e., from neutral position to 35 degrees plantar flexion), while the average human achilles tendon length is described as 180.6 ± 25 mm [28, 29]. In order to cover the typical surgical approach and to avoid errors of the lower leg volume due to coverage of too many muscle bellies at an ankle joint angle of 0 to 35 degrees, we decided to measure the volume 20 cm proximal to the malleolus fork. To cover the average size of the human tibial metaphysis, the ROI ankle was chosen at a location 5 cm proximal from the medial malleolus [30]. There exists a dearth of information concerning the volume variability between contralateral limbs in healthy subjects such as may be caused by leg dominance [31]. This study demonstrates no significant differences in the overall volume and the respective ROI between right and left leg in our study participants (Fig. 3). However, some limitations of this study need to be reflected. Despite a priory power analysis to determine the minimal sample size for this study, the small quantity of participants in this study may limit the conclusion relative to the population at large. However, previous studies showed, in similar small or even smaller sample sizes, a low mean percentage error using the same portable Artec 3D scanner [9, 24]. This study does not evaluate the circadian and environmental influences on the lower extremities’ soft tissue over time. It needs to be considered that different ankle joint angles may lead to a change in volume in the respective ROI, which is why it is imperative that measures were always made in the same position for both legs. The study population consists predominantly of healthy, white individuals. To study only one single population was not by design but rather a matter of coincidence and circumstances. That does not only impede the generalizability of our results but also might make them inapplicable to a nonwhite population or populations with diversity or ethnical background. This could lead to the perception of racial and ethnic disparities. Previous studies addressed such conflicts as clinical trials tend to offer far too little racial and ethnic diversity [32, 33].

Indeed, this method should also be applied within populations of different ethnical backgrounds in future studies. The proband population was chosen to be healthy and rather young, to reduce bias and normalize the values, making them comparable throughout the proband population. The comparison may be disturbed in patients with chronic venous insufficiency, injury to both lower extremities and other diseases that may be associated with swelling of the lower extremities. This study is intended to be considered a pilot study on healthy probands to make different decongestant management for soft tissue swelling objectively comparable in subsequent studies. When our standardization efforts are followed, the selected ROI are valid and reliable for assessing and comparing soft tissue swelling of injured limbs with the healthy contralateral side. Furthermore, scanning with the Artec Eva 3D Scanner for both lower extremities took 5.1± 2 min, which is faster than water displacement measurements, but slightly slower than conventional tape measurement [8]. But because of the more accurate method of evaluating soft tissue volume it was deemed the most suitable for this application. This fast and easy handled 3D scanner may be used in urgent or deferred situations where one is faced with critical or borderline soft tissue findings. The establishment of valid ROI in this pilot study form the basis for further studies to evaluate the clinical applicability of the scanner.

## Conclusion

This pilot study defines three regions of interest of the lower leg with no significant difference between the right and left side. Thus, bilateral comparison of the established ROI with a portable 3D Scanner (Artec 3D EVA) can be used as a valid and reliable instrument for quantifying shape and volume fluctuations of lower extremities in healthy probands. Based on these regions of interest, further studies with side- by- side comparison of uninjured and injured legs in patients are needed to evaluate the clinical applicability of the scanner. This may assist in formulating significant strategies for soft tissue management of severely injured lower extremities in clinical practice.

## Data Availability

All data generated or analysed during this study are included in this published article.

## Abbreviations

ROI: Regions of interest
3D: 3 dimensional
